# MicroRNAs in bovine adipogenesis: genomic context, expression and function

**DOI:** 10.1186/1471-2164-15-137

**Published:** 2014-02-18

**Authors:** Josue Moura Romao, Weiwu Jin, Maolong He, Tim McAllister, Le Luo Guan

**Affiliations:** 1 Department of Agricultural, Food and Nutritional Science, University of Alberta, Edmonton, Alberta, Canada; 2 Lethbridge Research Centre, Agriculture and Agri-Food Canada, Lethbridge, Alberta, Canada

**Keywords:** Adipogenesis, Adipose tissue, Bovine, Fat metabolism, Genomic context, microRNA, Cluster, Co-expression, Species specific

## Abstract

**Background:**

MicroRNAs (miRNAs) are small non-coding RNAs found to regulate several biological processes including adipogenesis. Understanding adipose tissue regulation is critical for beef cattle as fat is an important determinant of beef quality and nutrient value. This study analyzed the association between genomic context characteristics of miRNAs with their expression and function in bovine adipose tissue. Twenty-four subcutaneous adipose tissue biopsies were obtained from eight British-continental crossbred steers at 3 different time points. Total RNA was extracted and miRNAs were profiled using a miRNA microarray with expression further validated by qRT-PCR.

**Results:**

A total of 224 miRNAs were detected of which 155 were expressed in all steers (n = 8), and defined as the core miRNAs of bovine subcutaneous adipose tissue. Core adipose miRNAs varied in terms of genomic location (59.5% intergenic, 38.7% intronic, 1.2% exonic, and 0.6% mirtron), organization (55.5% non-clustered and 44.5% clustered), and conservation (49% highly conserved, 14% conserved and 37% poorly conserved). Clustered miRNAs and highly conserved miRNAs were more highly expressed (p < 0.05) and had more predicted targets than non-clustered or less conserved miRNAs (p < 0.001). A total of 34 miRNAs were coordinately expressed, being part of six identified relevant networks. Two intronic miRNAs (miR-33a and miR-1281) were confirmed to have coordinated expression with their host genes, transcriptional factor *SREBF2* and *EP300* (a transcriptional co-activator of transcriptional factor C/EBPα), respectively which are involved in lipid metabolism, suggesting these miRNAs may also play a role in regulation of bovine lipid metabolism/adipogenesis. Furthermore, a total of 17 bovine specific miRNAs were predicted to be involved in the regulation of energy balance in adipose tissue.

**Conclusions:**

These findings improve our understanding on the behavior of miRNAs in the regulation of bovine adipogenesis and fat metabolism as it reveals that miRNA expression patterns and functions are associated with miRNA genomic location, organization and conservation.

## Background

Adipose tissue biology is fundamental to functional physiology as fat is not only the major tissue of energy storage in mammals, but also an important endocrine organ [1,2]. Dysfunctions in adipose tissue metabolism have become an important health concern as obesity has reached epidemic proportions [3] with approximately a quarter of the world’s population being overweight or obese [4]. The consumption of high fat foods is clearly associated with obesity [5] and as a consequence both breeding and nutritional programs have focused on increasing the leanness of beef [6,7]. Meat is an important protein source and the world meat demand is projected to increase by 73% from 2010 to 2050 [8]. Fat is an important component in meat quality and impacts animal productivity [9,10]; therefore an understanding of the molecular regulation of adipogenesis is pivotal in the development of strategies to manipulate adiposity and improve beef quality.

Adipogenesis involves the development of mature adipocytes from preadipocytes [11] and is responsible for modulating adiposity in individuals. Adipogenesis is genetically regulated and studies have shown the importance of adipogenic transcription factors (PPARγ, C/EBPs, KLFs and SERBP) in fat development as they regulate the expression of adipogenic genes involved in adipocyte differentiation [12,13]. However, the regulatory mechanisms of microRNAs (miRNAs) in adipose tissue are not well understood. miRNAs are small non-coding RNAs composed of approximately 22 nucleotides that repress gene expression by binding to messenger RNAs in a sequence-specific manner [14]. In 2004, miR-143 was the first miRNA reported to affect differentiation of human adipocytes [15]. Since then, several studies in humans and mice have shown that miRNAs regulate adipogenesis in a pro or anti-adipogenic manner [16,17] through repression of various genes, including transcriptional factors such as *PPARγ*, *PPARα* and *KLF5 *[18-21] or through the regulation of pathways that impact adipogenesis such as WNT signalling [22]. However, few miRNAs had their targets identified, such as miR-130 that regulates *PPARγ* and inhibits adipogenesis [23], and miR-181a that regulates TNFα and increases adipogenesis [24]. Studies using bovine adipose tissue have shown that miRNA expression profiles change according to internal and external environmental factors such as subcutaneous fat thickness [25], fat depot (subcutaneous vs. visceral fat) and dietary manipulation (high vs. low fat content) [26]. However, it is not clear how miRNA expression and function are impacted by the compositional structure of DNA that surrounds miRNA genes, also known as genomic context characteristics. These might include features such as the distance between miRNA genes, which has been suggested to be a determinant for coordinated miRNA expression [27] or the location of miRNA genes in relation to protein coding genes (intergenic, intronic, exonic, or mirtron). The elucidation of these aspects may provide important clues with regard to the regulation and function of miRNAs in bovine adipogenesis. Therefore, this study aimed to determine how genomic context of miRNA genes and their conservation are associated with the expression and function in bovine subcutaneous adipose tissue.

## Results

### Genomic organization of bovine miRNAs

**Figure 1:**
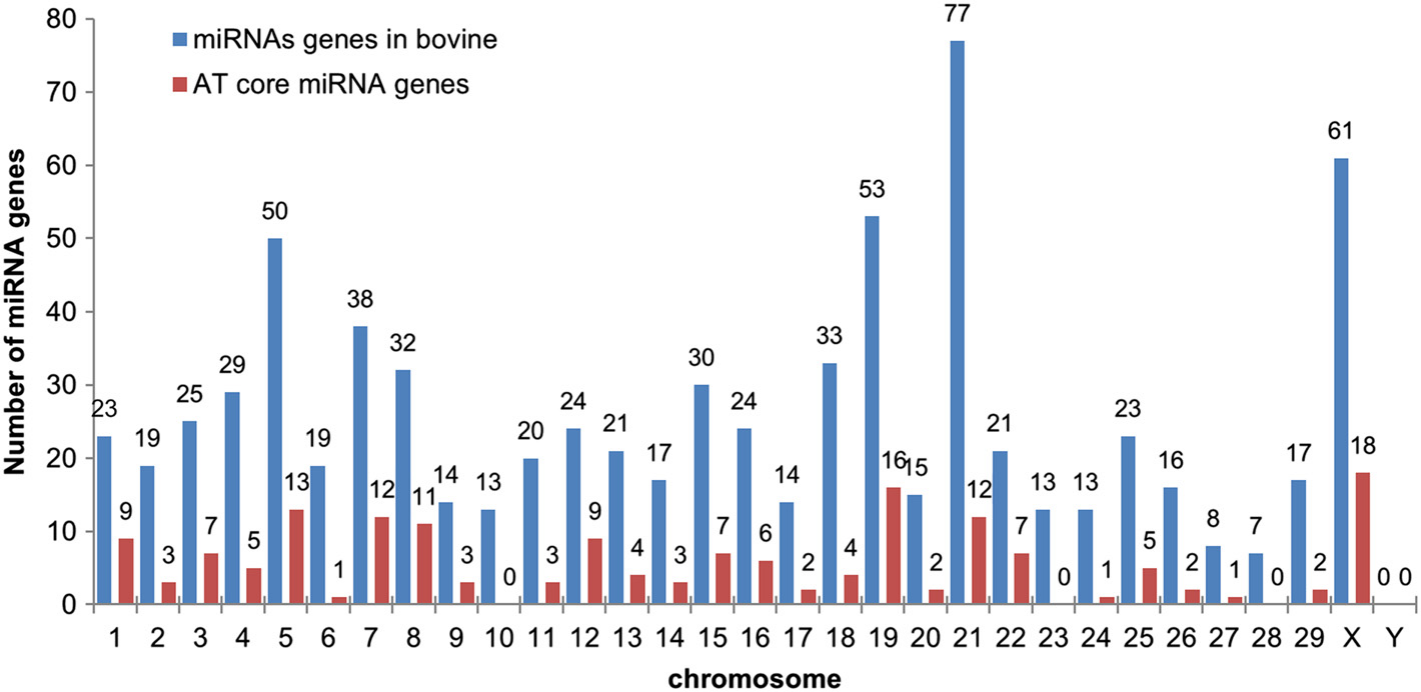
**Number of miRNA genes in each chromosome.** AT core miRNAs consist of miRNAs detected in the subcutaneous adipose tissue of all steers.

The bovine genome is organized in 29 pairs of autosomes and 2 sex chromosomes. A total of 755 mature miRNAs have been detected in the bovine and originate from 769 miRNAs genes coded in different genomic loci of virtually every bovine chromosome, except the sex chromosome Y (miRBase, release 19). It is worth mentioning that the difference in the quantity of miRNAs and miRNA genes happens because their relationship is not one to one, as some miRNA genes may create more than one miRNA (eg. microRNA 151 gene originates miR-151-3p and miR-151-5p) and some miRNAs can originate from different miRNA genes (eg. miR-378 from miRNA 378 genes on chromosomes 4 and 7). Based on miRNA microarray analysis, approximately 30% of the known bovine miRNAs (n = 224) were detected in subcutaneous adipose tissue of which 155 miRNAs were expressed in the subcutaneous fat of all steers (n = 8) in at least one time point, being defined as the adipose tissue core miRNAs (AT core miRNAs) in this study even if their expressions may be not adipose tissue specific. The genomic distribution of AT core miRNAs was highly variable as some chromosomes (chr 10, chr 23, chr 28, and chr Y) possessed no adipose core miRNA genes while others coded for several, such as chromosome X which had 18 of them (Figure 1).

**Figure 2:**
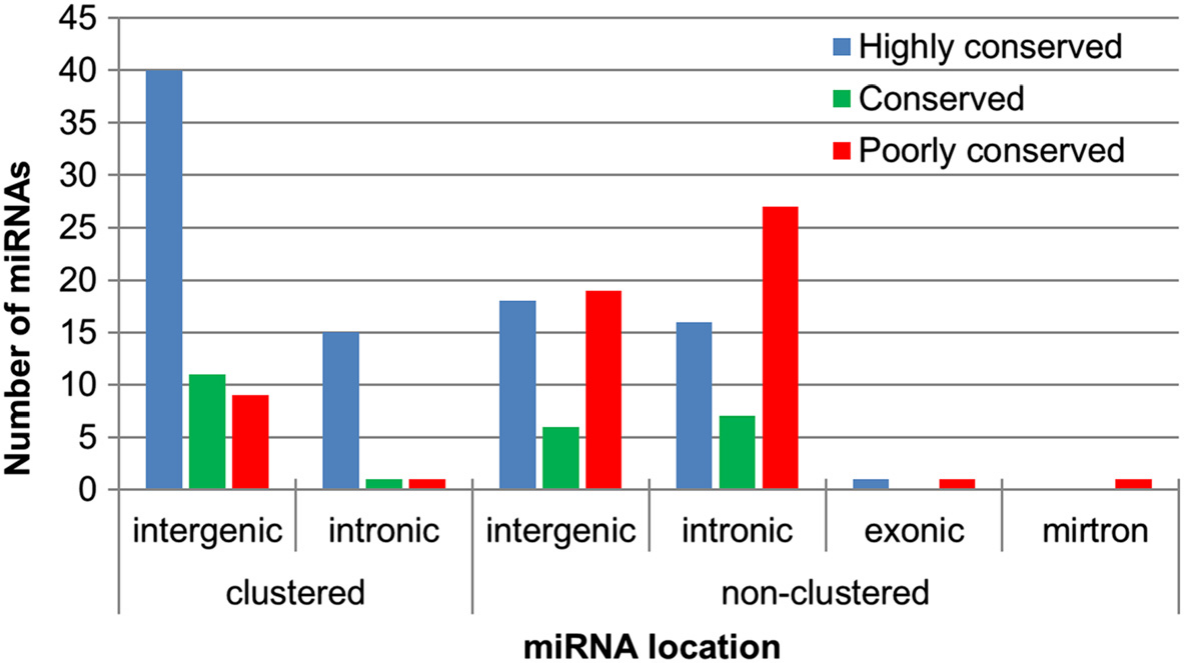
**Genomic context and conservation of AT core miRNAs.** AT core miRNAs consist of miRNAs detected in the subcutaneous adipose tissue of all steers.

Mature miRNAs (n = 155) expressed in all steers were classified into three main conservation categories: 76 highly conserved miRNAs (conserved across most vertebrates), 22 conserved miRNAs (conserved across most mammals), and 57 poorly conserved miRNAs (conserved not beyond placental mammals) (TargetScan http://www.targetscan.org/cgi-bin/targetscan/mirna_families.cgi?db=vert_61). The genomic context of these miRNAs was also investigated in terms of organization (clustered and non-clustered) and their location as intergenic, intronic, exonic, or mirtron as shown in Figure 2. miRNAs in clusters represented only 26.7% of all bovine miRNAs (n = 755); however, that percentage increased to 44.5% when only AT core miRNAs (n = 155) were considered, an indication of their role in the core regulation of bovine adipose tissue. The organization of miRNAs in clusters was associated to conservation status as the highly conserved miRNAs were by far the most numerous, representing an overlap of 71.4% (Figure 2).

### miRNA expression in adipose tissue

**Figure 3:**
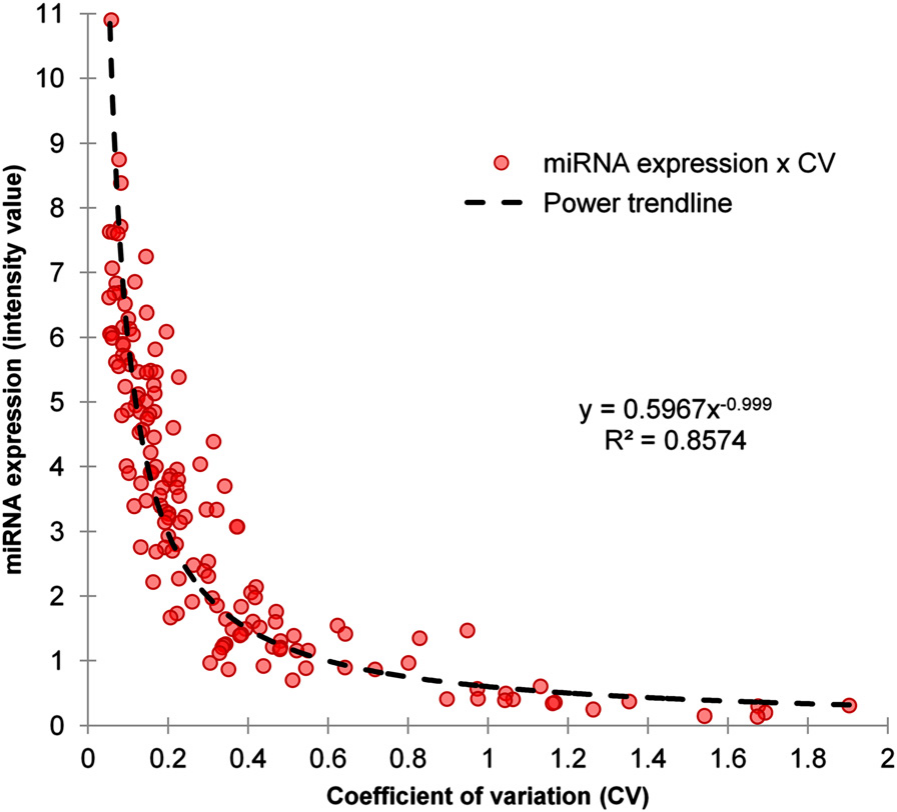
**AT core miRNAs: miRNA average expression vs. coefficient of variation.** Each circle represents one unique miRNA and the coordinates of X and Y axis are the respective values for average expression of the miRNA and its coefficient of variation from different samples. AT core miRNAs consist of miRNAs detected in the subcutaneous adipose tissue of all steers.

Expression of AT core miRNAs (n = 155) varied widely, as microarray intensity values (normalized) ranged from close to zero (miR-2425-3p) to up to 10.9 (miR-2478). A power trendline illustrated the predictability of miRNAs behavior in terms of average expression and variation among samples (CV) with the equation y = 0.5967x^-0.999^, (R^2^ = 0.857) where “y” represents miRNA expression and “x” is the CV (Figure 3). miRNA expression among steers varied considerably with type of miRNA, ranging from highly uniform such as miR-26a with a coefficient of variation (CV) of 0.05 to highly variable such as miR-2428 with a CV of 1.90 (Figure 3). The average expression of miRNAs and their coefficient of variation were negatively correlated (R = -0.71) as highly expressed miRNAs tended to occur consistently among steers while poorly expressed miRNAs varied widely among individuals.

**Figure 4:**
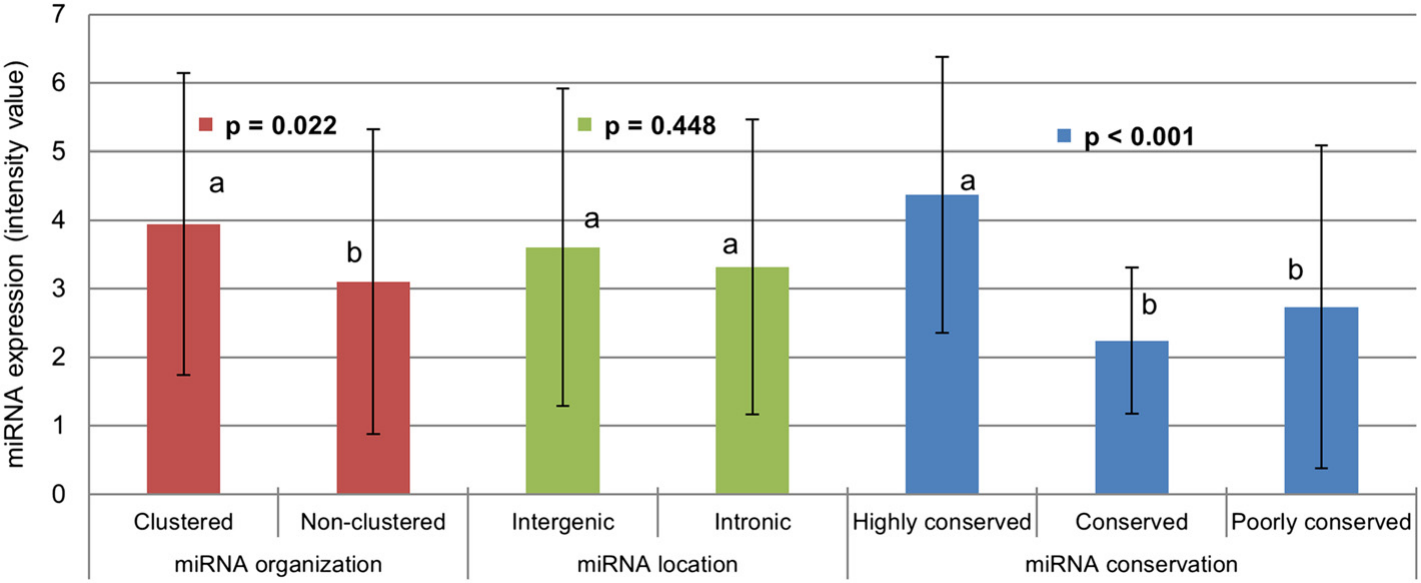
**Expression of AT core miRNAs according to miRNA gene organization, location and conservation.** Columns with different letters **(a,b)** differ statistically within each category comparison (p < 0.05). AT core miRNAs consist of miRNAs detected in the subcutaneous adipose tissue of all steers.

The genomic context in which AT core miRNAs genes are located seems to be a factor impacting expression, as clustered miRNAs were more highly expressed than unclustered miRNAs (p = 0.022). However, being located in an intron of a protein coding gene (intronic miRNA) or being an independent transcription unit in between genes (intergenic miRNA) did not seem to impact global miRNAlevels (p = 0.448). miRNAs that were highly conserved were expressed more (p < 0.001) than miRNAs moderately conserved or poorly conserved (Figure 4). Expression of miRNAs also varied considerably within each category according to miRNA gene locations, and with degree of conservation. For instance, miRNAs that were poorly conserved could still be highly expressed, as was observed with miR-2478 (a bovine specific miRNA) which was non-clustered and poorly conserved, but the most highly expressed miRNA in bovine adipose tissue (Additional file 1).

### Predicted targets of AT core miRNAs

**Figure 5:**
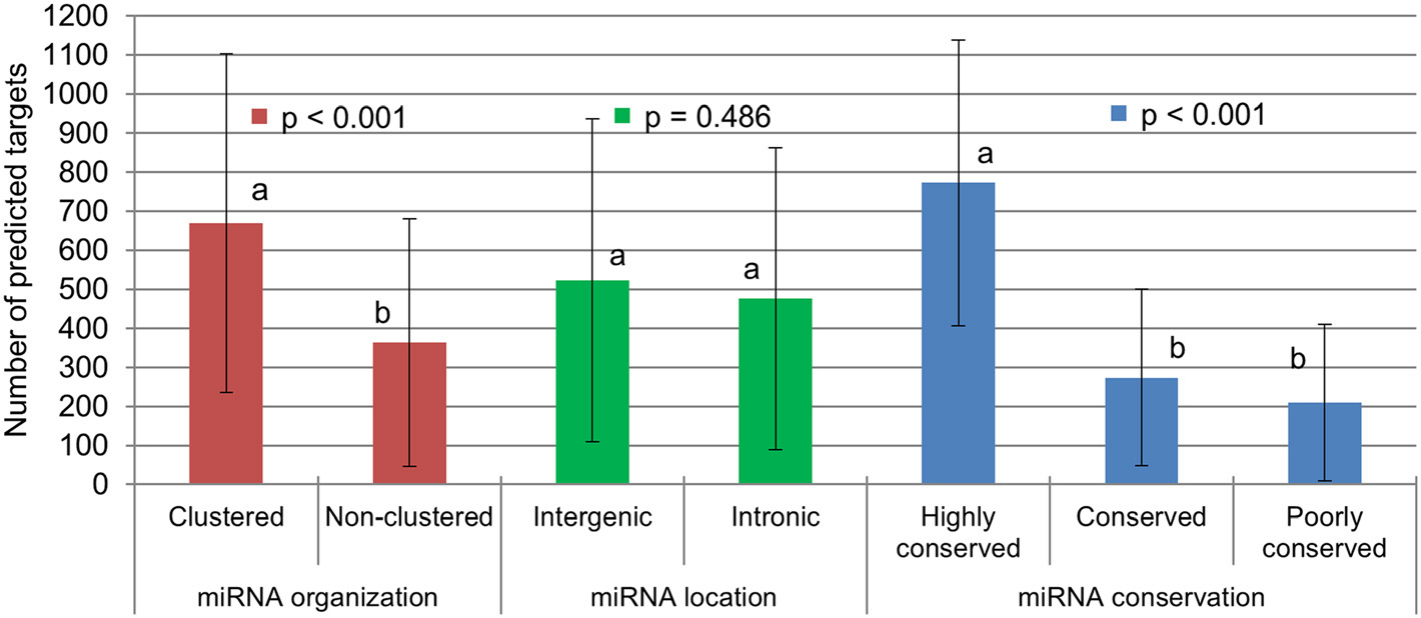
**Number of predicted targets according to AT core miRNA organization, location and conservation.** Columns with different letters **(a,b)** differ statistically within each category comparison (p < 0.05). AT core miRNAs consist of miRNAs detected in the subcutaneous adipose tissue of all steers.

AT core miRNAs had on average 500 predicted targets by TargetScan 6.2 (http://www.targetscan.org/), ranging from 3 target genes for miR-2892 and 1262 targets predicted for miR-30a-5p, miR-30b-5p, miR-30c, miR-30d, and miR-30f. The number of predicted targets varied (p < 0.001) with genomic organization and conservation, but the location of miRNAs had no (p > 0.05) relationship to number predicted targets (Figure 5). On average, highly conserved miRNAs had three times more the number of predicted targets as compared to poorly conserved miRNAs, while clustered miRNAs had 1.8 fold more predicted targets than non-clustered miRNAs.

### Co-expression patterns of AT core miRNAs

**Figure 6:**
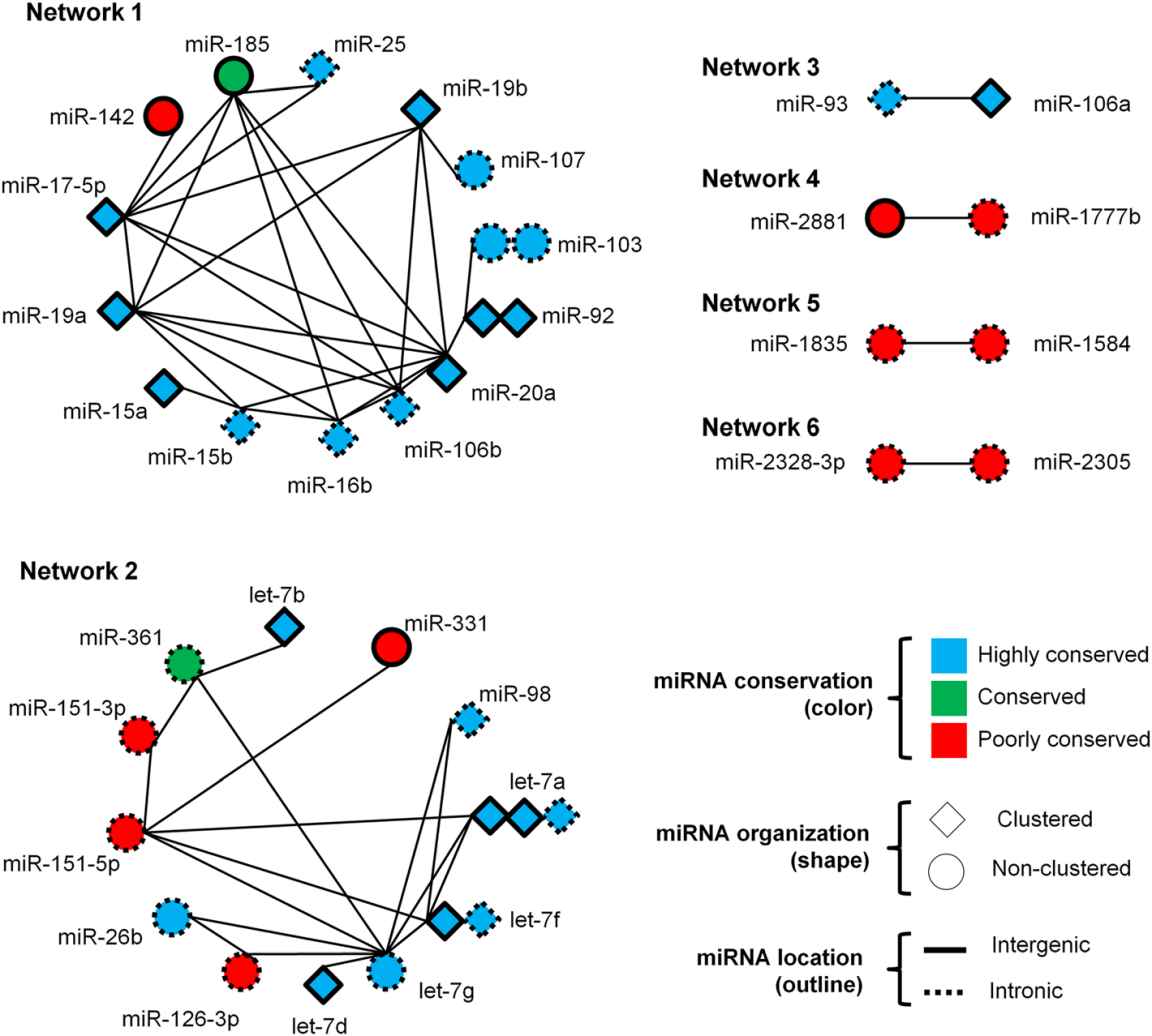
**Relevant networks of AT core miRNAs.** miRNAs with connecting lines have highly correlated (R^2^ > 0.95) expression patterns. miRNAs represented by multiple shapes indicates that they are coded at more than one genomic location. AT core miRNAs consist of miRNAs detected in the subcutaneous adipose tissue of all steers.

**Figure 7:**
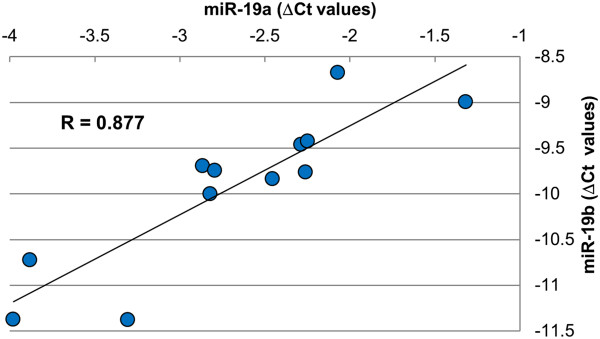
**Expression levels of miR-19a and miR-19b by qRT-PCR.** Δ Ct values were calculated as follow: ΔCt miR-19a = Ct miR-19a – Ct miR-181a and ΔCt miR-19b = Ct miR-19b – Ct miR-181a.

The relationship among the expressions of AT core miRNAs across adipose tissue samples was either positively correlated, non-correlated or negatively correlated. For example, the levels of miR-20a and miR-106b were positively correlated (R = 0.99) while miR-2288 and miR-671 were negatively correlated (R = -0.76). Relevant networks analysis revealed bovine miRNAs with coordinated expression that were likely to participate in common biological processes in adipose tissue. miRNA networks were generated by examining those miRNAs that exhibited highly correlated (R^2^ > 0.95) expression over the 24 adipose tissue samples (Figure 6). A total of six relevant networks composed of 34 miRNAs were generated, with 14 miRNAs in Network 1, 12 in Network 2 and 2 miRNAs in each of networks 3, 4, 5, and 6. The majority of miRNAs (70%) involved in the large networks 1 and 2 were highly conserved; while most miRNAs (6 out of 8) from small networks 3, 4, 5, and 6 were poorly conserved (Figure 6). Several miRNAs in the relevant networks were members of bovine miRNA clusters: miR-17-5p, 19a, 20a, 19b and 92 (cluster 17 ~ 92); miR-25 and miR-106b (cluster 106b ~ 25); miR-16b and 15b (cluster 16b ~ 15b) and miR-15a (cluster 16a ~ 15a) in Network 1. While in Network 2 let-7a and let-7b were members of the let-7a ~ let-7b cluster; let-7a, let-7d, and let-7f formed the let-7a ~ let-7d cluster and let-7f and miR-98 were members of the 98 ~ let-7f cluster. Interestingly, let-7a and let-7f were present in more than one cluster as each of them is coded by more than one miRNA gene located on different chromosomes. miRNA pairs in networks 3, 4, 5 and 6 were highly correlated but, their miRNA genes were not organized in clusters. For a full description of all miRNA clusters expressed in adipose tissue see Additional file 1. A frequency histogram of all pairwise correlations among miRNAs is shown in Additional file 2 and a hierarchical dendogram clustering the expression of all 155 AT core miRNAs is shown in Additional file 3. Quantitative RT-PCR confirmed (p < 0.001) the correlated expression of miR-19a and miR-19b in Network 1 and supported the microarray findings of coordinated expression among miRNAs that are members of the same network (Figure 7).

### AT core miRNAs hosted in protein coding genes

**Figure 8:**
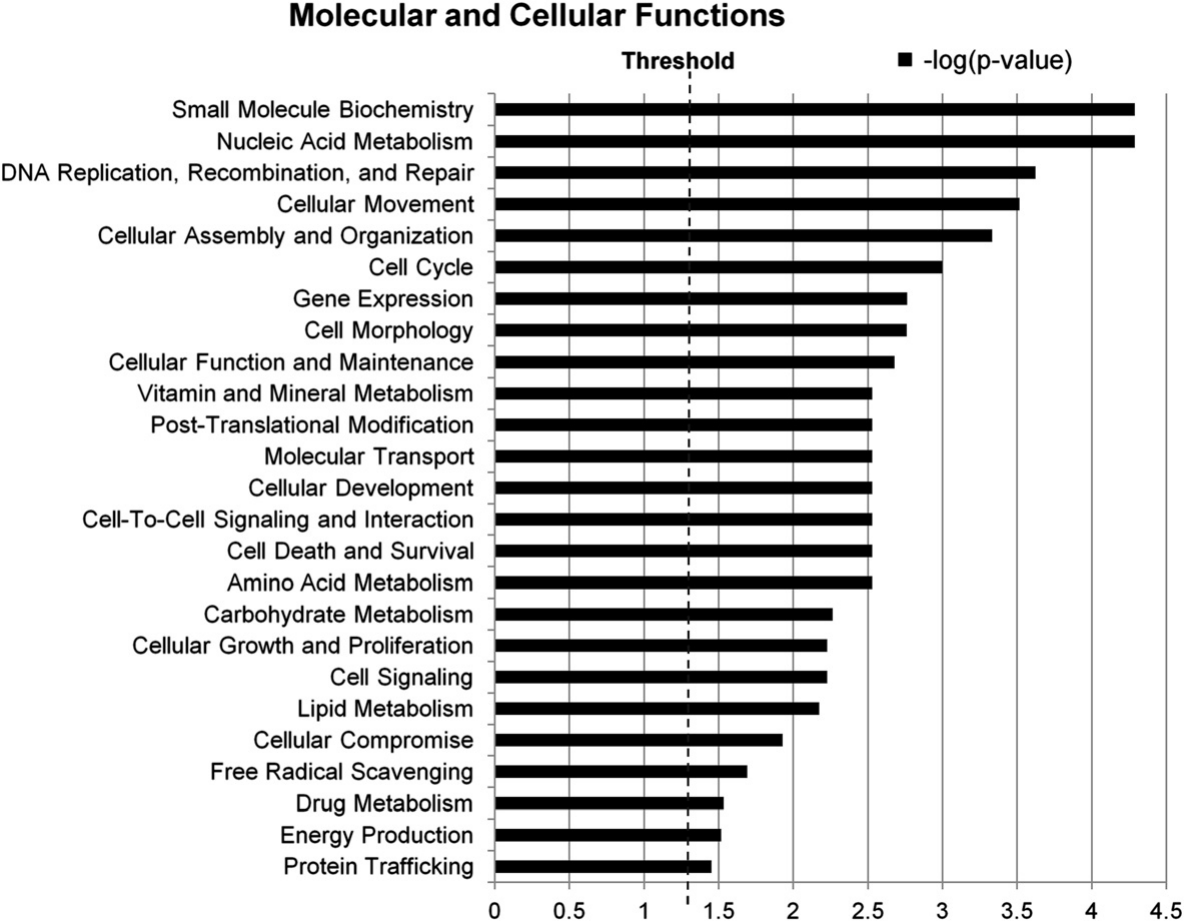
**Molecular and cellular functions of protein coding genes that host AT core miRNAs.** The likelihood of the association between the genes and a biological function is represented as –log(p-value), with larger bars being more significant than shorter bars. The vertical line indicates the cutoff for significance (p-value of 0.05). AT core miRNAs consist of miRNAs detected in the subcutaneous adipose tissue of all steers.

A total of 60 AT core miRNAs were located inside 60 protein coding genes. Most of them were intronic (n = 57), two were exonic and one was a mirtron. It is important to note that some of these genes host more than one miRNA such as minichromosome maintenance complex component 7 (*MCM7*) which contains miR-106b, 93 and 25. Also, some miRNAs are inside introns of more than one gene as is the case for miR-103 within the introns of pantothenate kinase 2 (*PANK2*) and pantothenate kinase 3 (*PANK3*). Therefore the biogenesis of intronic miRNAs does not always follow the rule of a host gene originating only one intronic miRNA or an intronic miRNA being generated by only one host gene. Intronic miRNAs and their host genes may function in common in the same pathways as their expression might be coordinated. Therefore, functional analysis of genes possessing intronic miRNAs may reveal the biological functions of their miRNAs. IPA® software (http://www.ingenuity.com/) mapped 54 out of 60 genes hosting AT core miRNAs to the Ingenuity Knowledge Base revealing that these miRNAs are hosted by genes coding for kinases (n = 6), phosphatases (n = 2), other enzymes (n = 8), ion channel proteins (n = 2), transcription regulators (n = 7), a translation regulator (n = 1), transporters (n = 4), and other proteins (n = 24). These genes were further submitted to a Core analysis (functional analysis) that showed that 41 out of the 54 genes were associated (p < 0.05) with 25 categories of IPA molecular and cellular functions (Figure 8). The other 13 genes were not associated with IPA functions.

Among the significant functions, several were related to general cellular metabolism such as small molecule biochemistry, cellular movement, cellular assembly and organization, cell cycle among others. A total of five genes were associated with lipid metabolism: E1A binding protein p300 (*EP300*), peroxisome proliferator-activated receptor gamma, coactivator 1 beta (*PPARGC1B*), platelet-derived growth factor receptor, beta polypeptide (*PDGFRB*), protein tyrosine kinase 2 (*PTK2*), and sterol regulatory element binding transcription factor 2 (*SREBF2*) (Table 1). Each of these genes hosted a single miRNA and a functional analysis indicated that these five genes were involved together in several aspects of lipid metabolism (Table 1).

**Table 1:** Functional roles of genes hosting adipose core miRNAs in lipid metabolism

**Functions annotation**	**p-Value**	**Molecules**
Concentration of lipid	5.72E-07	*EP300* (miR-1281), *PDGFRB* (miR-1777b), *PPARGC1B* (miR-378), *PTK2* (miR-151-5p), *SREBF2* (miR-33a)
Concentration of phosphatidic acid	4.74E-06	*EP300* (miR-1281), *PDGFRB* (miR-1777b), *PTK2* (miR-151-5p)
Quantity of phosphoinositide	1.59E-04	*PDGFRB* (miR-1777b), *PTK2* (miR-151-5p)
Quantity of myristic acid	9.03E-04	*PPARGC1B* (miR-378)
Quantity of sn-glycero-3-phosphocholine	9.03E-04	*EP300* (miR-1281)
Oxidation of lipid	1.47E-03	*PPARGC1B* (miR-378), *SREBF2* (miR-33a)
Concentration of palmitic acid	1.81E-03	*PPARGC1B* (miR-378)
Concentration of stearic acid	1.81E-03	*PPARGC1B* (miR-378)
Concentration of cholesterol	2.98E-03	*PPARGC1B* (miR-378), *SREBF2* (miR-33a)
Quantity of phosphatidylinositol-3-phosphate	3.01E-03	*PDGFRB* (miR-1777b)
Storage of cholesterol	4.51E-03	*SREBF2* (miR-33a)
Uptake of palmitic acid	4.51E-03	*PPARGC1B* (miR-378)
Metabolism of membrane lipid derivative	5.82E-03	*PDGFRB* (miR-1777b), *SREBF2* (miR-33a)
Quantity of phosphatidylinositol-3,4,5-triphosphate	9.00E-03	*PTK2* (miR-151-5p)
Synthesis of phosphatidylinositol-3,4,5-triphosphate	1.05E-02	*PDGFRB* (miR-1777b)
Oxidation of palmitic acid	1.20E-02	*PPARGC1B* (miR-378)
Accumulation of cholesterol	1.47E-02	*SREBF2* (miR-33a)
Synthesis of cholesterol	1.73E-02	*SREBF2* (miR-33a)
Synthesis of lipid	2.15E-02	*PDGFRB* (miR-1777b), SREBF2 (miR-33a)
Homeostasis of cholesterol	2.36E-02	*SREBF2* (miR-33a)
Hydrolysis of phosphatidylinositol	2.74E-02	*PDGFRB* (miR-1777b)

E1A binding protein p300 (*EP300*), peroxisome proliferator-activated receptor gamma, coactivator 1 beta (*PPARGC1B*), platelet-derived growth factor receptor, beta polypeptide (*PDGFRB*), protein tyrosine kinase 2 (*PTK2*), and sterol regulatory element binding transcription factor 2 (*SREBF2*).

The relationship between genes involved in lipid metabolism and their intronic miRNAs was further analyzed by comparing their expression using qRT-PCR. The analysis included *EP300* (miR-1281), *PPARGC1B* (miR-378), *PTK2* (miR-151-5p) and *SREBF2* (miR-33a). The expression of the pair *PDGFRB* (miR-1777b) was not assessed as it was not possible to design primers for miR-1777b owing to its very high GC content. A Pearson correlation analysis showed that expression of miR-1281 and miR-33a was correlated (p < 0.05) with their host gene, respectively, while no correlation (p > 0.05) with the host gene was observed for miR-378 and miR-151-5p (Table 2).

**Table 2:** Correlation analysis of host genes and intronic miRNAs expression by qRT-PCR

**Gene**	**miRNA**	**R**	**p-value**
*EP300*	miR-1281	0.624	0.030
*PPARGC1B*	miR-378	0.193	0.554
*SREBF2*	miR-33a	0.635	0.027
*PTK2*	miR-151-5p	0.001	0.996

E1A binding protein p300 (EP300), peroxisome proliferator-activated receptor gamma, coactivator 1 beta (PPARGC1B), sterol regulatory element binding transcription factor 2 (SREBF2) and protein tyrosine kinase 2 (PTK2). Correlation was significant at p < 0.05.

### Bovine specific miRNAs

Among the 57 poorly conserved AT core miRNAs, a total of 23 had seed regions (7 nucleotides at the 5′ end of miRNA) that are found only in bovine (Table 3). Three bovine specific miRNAs (miR-1584, miR-2412 and miR-2374) had different sequences; however, they shared the same seed region “UGGGGCU”. Except for miR-425-5p, all bovine specific miRNAs did not cluster and 13 out of 23 were located in intergenic regions. Expression of bovine specific miRNAs varied considerably and was as much as 70 fold higher for miR-2478 than that of miR-2882. Similarly, the numbers of predicted targets for each bovine specific miRNA were highly variable (Table 3).

**Table 3:** Bovine specific miRNAs expressed in adipose tissue of all steers

**microRNAs**	**Chromosome**	**Cluster**	**Location**	**Host gene**^**1**^	**miRNA expression**	**Predicted targets**^**2**^	**Seed region**^**3**^
bta-miR-2478	chr9	no	intergenic	-	10.90 ± 0.63	116	UAUCCCA
bta-miR-126-3p	chr11	no	intronic	*EGFL7*	7.71 ± 0.63	26	GUACCGU
bta-miR-2305	chr13	no	intronic	*RIN2*	6.09 ± 1.19	109	GGGGGUG
bta-miR-2328-3p	chr18	no	intronic	*ZNF821*	5.39 ± 1.22	144	CCCCCUC
bta-miR-1584	chr3	no	intronic	*TAGLN2*	5.13 ± 0.85	135	UGGGGCU
bta-miR-2888	chr21	no	intergenic	*-*	4.60 ± 0.98	93	GUGGGGU
bta-miR-199c	chr19	no	intronic	*NUP88*	4.01 ± 0.38	704	ACAGUAG
bta-miR-2881	chr7	no	intergenic	*-*	3.95 ± 0.88	59	GGGCGGG
bta-miR-2332	chr19	no	intronic	*UTP6*	3.39 ± 0.39	392	GGUUUAA
bta-miR-2412	chr3	no	intergenic	*-*	3.34 ± 0.99	135	UGGGGCU
bta-miR-2455	chr7	no	intergenic	*-*	3.07 ± 1.14	413	CUGUGCU
bta-miR-2316	chr15	no	intergenic	*-*	2.53 ± 0.76	8	CUCCGGC
bta-miR-2374	chr22	no	intergenic	*-*	1.98 ± 0.83	135	UGGGGCU
bta-miR-2483	chrX	no	intergenic	*-*	1.62 ± 1.12	381	AACAUCU
bta-miR-2474	chr8	no	intronic	*SHB*	1.61 ± 0.96	5	ACCGGGC
bta-miR-425-5p	chr22	yes	intergenic	*-*	1.61 ± 0.66	74	UGACACG
bta-miR-2892	chr12	no	intergenic	*-*	1.52 ± 0.65	3	GCGACGG
bta-miR-1434	chr7	no	intronic	*EEF2*	1.02 ± 0.57	1134	AAGAAAU
bta-miR-2391	chr26	no	intergenic	*-*	0.55 ± 0.40	2732	AAAAAAA
bta-miR-2898	chr8	no	intergenic	*-*	0.37 ± 0.50	134	GGUGGAG
bta-miR-2424	chr5	no	intronic	*NCAPD2*	0.29 ± 0.49	225	GAUCUUU
bta-miR-2885	chr29	no	intergenic	*-*	0.20 ± 0.34	27	GGCGGCA
bta-miR-2882	chr7	no	exonic	*SMARCA4*	0.15 ± 0.23	19	GCCCGGG

^1^EGF-like-domain, multiple 7 (*EGFL7*), Ras and Rab interactor 2 (*RIN2*), zinc finger protein 821 (*ZNF821*), transgelin 2 (*TAGLN2*), nucleoporin 88 kDa (*NUP88*), UTP6, small subunit (*SSU*) processome component, homolog (yeast) (*UTP6*), Src homology 2 domain containing adaptor protein B (*SHB*), eukaryotic translation elongation factor 2 (*EEF2*), uncharacterized LOC509171 (*NCAPD2*), SWI/SNF related, matrix associated, actin dependent regulator of chromatin, subfamily a, member 4 (*SMARCA4*).

^2^The amount of targets by miRNA is based on TargetScan prediction tool release 6.2.

^3^Includes 7 nucleotides at positions 2–8 starting at the 5′end of the mature miRNA sequence.

**Figure 9:**
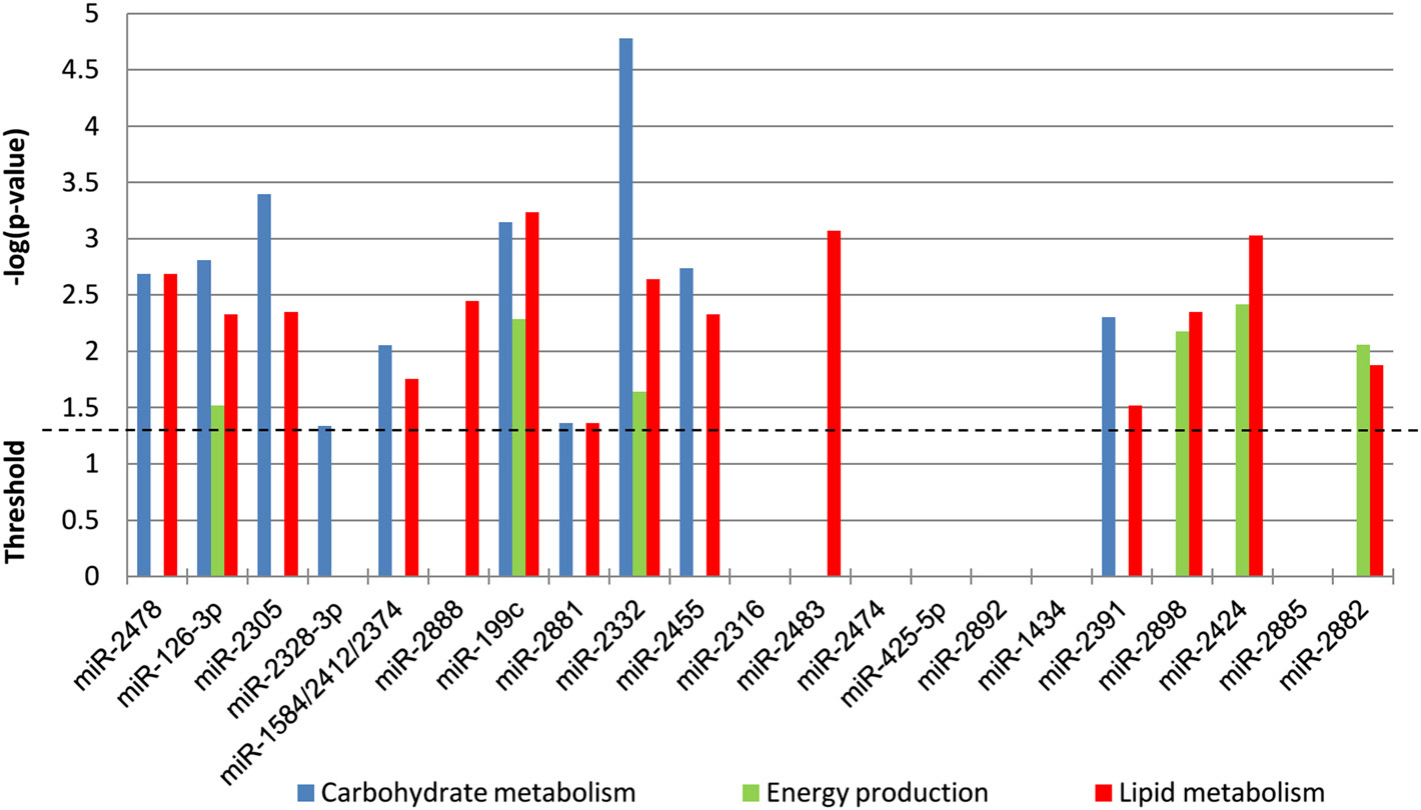
**Association of predicted targets from bovine specific miRNAs to energy production, carbohydrate, or lipid metabolism.** The likelihood of the association between the genes and a biological function is represented as –log(p-value), with larger bars being more significant than shorter bars. The vertical line indicates the cutoff for significance (p-value of 0.05). AT core miRNAs consist of miRNAs detected in the subcutaneous adipose tissue of all steers.

The potential regulatory functions of the 23 bovine specific miRNAs expressed in all cattle were investigated by performing individual target prediction analyses (TargetScan) [28]. Only targets predicted with total context^+^ scores ≤ -0.3 were selected to reduce false positive predictions [29]. As miR-1584, miR-2412 and miR-2374 have the same seed region, they were analyzed as one entry “miR-1584/2412/2374”. An IPA® Core Analysis was performed on predicted targets of each bovine specific miRNA in order to identify their function and elucidate the potential regulatory roles of these miRNAs. In combination, the 23 core adipose bovine specific miRNAs were predicted to regulate genes associated (p < 0.05) with 30 different molecular and cellular functions (Additional file 4). Fifteen of these were potential regulators of pathways related to lipid metabolism, carbohydrate metabolism and/or energy production as one or more of their predicted targets were involved (p < 0.05) with these functions (Figure 9). Additionally, these 15 miRNAs were also associated with predicted targets involved in other functions including cell-to-cell signalling and interaction. miR-2892 was the only bovine specific miRNA that was not associated with any predicted molecular and cellular function.

## Discussion

Knowledge of miRNA biology has been growing consistently over the past few years, but a clear understanding of their role in the regulation of cellular metabolism remains elusive. miRNAs are a group of diverse regulatory molecules with potential to target hundreds of genes [30] and integrate complex molecular regulatory networks. Studies have focused on identifying differential expression of miRNAs in adipose of individuals submitted to opposite conditions such as low fat vs. high fat diet treatments [31] or lean vs. obese individuals [32] which reveals only particular aspects of miRNA biology in adipose tissue. However, in this study we aimed to perform a more holistic approach by focusing on the genomic context features of miRNA genes and their global expression under physiological adipose tissue conditions.

The genome of animals is mostly non-protein-coding and evidence suggests that the amount of non-coding DNA and non-coding RNA transcribed from it correlates well with the biological complexity of organisms in the tree of life [33]. To date, the amount of miRNAs identified in bovine (n = 755) is the third highest in mammals following to those of human (n = 2042) and mice (n = 1281) [34]. Bovine miRNAs are not evenly distributed within the genome as numbers may vary up to 10 fold among individual chromosomes (Figure 1). Chromosome X of several mammalian species has been reported to have a higher miRNA density (# miRNA/chromosome size) as compared to those associated with autosomes [35]. Interestingly, chromosome X had the highest number of AT core miRNAs expressed in our study, suggesting a relevant role of this chromosome and its miRNAs in the function of adipose tissue. This variation in distribution may contribute to the differences in fat metabolism observed between male and female mammals in a manner analogous to the proposed role that miRNAs on chromosome X have on immune functions between female vs. male in humans and mice [36]. No bovine miRNA gene was identified on Chromosome Y. This could reflect the fact that the reference bovine genome (UMD3.1) was generated from a cow. However, it is expected that few miRNAs are coded in the bovine chromosome Y as other mammals such as mice have none (0/1281 miRNAs) and humans only have 2 out of 2042 miRNAs coded on chromosome Y [34].

A total of 155 miRNAs were consistently detected in samples of subcutaneous adipose tissue from all individuals. However, it is probable that these miRNAs are not exclusively associated with adipocytes as adipose tissue is also composed of preadipocytes, macrophages, endothelial cells and stem cells. Therefore the results from this study reflect the possible role of miRNA in the regulation of adipose tissue as a whole. The core adipose miRNAs were heterogeneous in terms of genomic context and conservation status. A large portion (44.5%) of the miRNAs that integrated the core adipose miRNAs was organized in clusters (Figure 2). They differed from non-clustered miRNAs due to their close proximity on the genome (< 10 kb), indicating that they may be subjected to coordinated expression, by sharing the same promoter [37]. Interestingly, adipose core miRNAs that organized in clusters were both from the same (e.g. cluster including let-7a, -7d, -7f in chr. 8) or different miRNA families (e.g. cluster including miR-17-3p, -18a, -19a, -20a, -19b, -92 in chr. 12). The presence of miRNAs from different families in the same cluster suggests that coordinated expression of these miRNAs enables regulation of different target messenger RNAs, and consequently to control expression of different genes within a common pathway [38,39]. Adipose core miRNAs were coded mainly in intergenic regions (59.5%); however, intronic miRNAs were also numerous (38.7%). miRNAs encoded in intronic regions are generally co-expressed with the host gene and may represent an auto-regulatory mechanism [40]. They may also support host gene expression by either down-regulating antagonist genes or fine-tuning the expression of genes (miRNA targets) that co-operate with the host gene in specific biological processes [41]. Exploring the functions of genes hosting intronic miRNAs may provide insight into the role of these molecules in bovine adipose tissue.

The behaviour of core adipose miRNAs followed a trend in which expression was inversely correlated to miRNA variation among different samples. miRNAs highly expressed had more consistent levels among samples, while miRNAs lowly expressed were more variable (Figure 3) which resembles the behaviour of genes as observed in a study that profiled the expression of 11,335 transcripts in lymphoblastoid cell lines from 210 humans [42]. The nature of the function performed by each gene is likely to influence the level of expression variation in the population [43], which might also occur to miRNAs. In addition, highly conserved miRNAs expressed higher than poorly conserved miRNAs (Figure 4). This result supports the idea that consistently high expressed miRNAs play critical roles in the regulation of cellular processes in bovine adipose tissue as they tend to be maintained by natural selection in most vertebrates (highly conserved miRNAs). This idea is also corroborated by the fact that highly expressed miRNAs are under intense selective pressure to maintain sequence uniformity while lowly expressed miRNAs tend to have much lower selection pressure being more prone to evolve rapidly [44]. Conservation status of bovine miRNAs was also associated to the amount of targets predicted by TargetScan (Figure 5) as highly conserved miRNAs had approximately three times more targets than poorly conserved miRNAs. Although computational tools such as TargetScan are powerful predictors of miRNA targets, they may generate false positive results. Our results are supported by a human study that reported that highly conserved miRNAs had higher repression efficiency (repression level and amount of targets) as compared to less conserved miRNAs [45]. And that might be partially influenced by the fact that in general highly conserved miRNAs are higher expressed then less conserved miRNAs. The behavior of highly conserved miRNAs was similar to that of clustered miRNAs in terms of expression (Figure 4) and amount of predicted targets (Figure 5). This is explained by the fact that highly conserved miRNAs tended to be organized in clusters, while less conserved miRNAs were more unclustered. The association between miRNA conservation and miRNA genomic organization was evident when miRNA relevant networks analysis was performed (Figure 6).

It is challenging to determine the scope of miRNA regulation in a biological process as each miRNA may target several mRNAs and each mRNA may be regulated by multiple miRNAs [46]. Evidence shows that miRNAs may work in a coordinated fashion to regulate gene expression in complex networks [47] and co-expressed miRNAs might be part of mechanisms of functional redundancy and cooperation [48]. For example, a study has shown that miR-370 regulated the expression of lipogenic genes indirectly by controlling the expression of miR-122 [49]. However, identifying miRNA functional networks is difficult as computational prediction tools are not accurate enough to identify only the true miRNA targets. Therefore, relying on miRNA expression data is an important piece of information to determine which miRNAs are functioning in a coordinated manner to regulate a certain biological process. Our study identified six relevant miRNA networks using miRNA expression data, suggesting that miRNAs regulate gene expression in bovine adipose tissue in a coordinated fashion. Although it is not possible to define the exact roles of each miRNA network identified in adipose tissue, some of the miRNAs identified have already been studied. For example miR-17-5p, 19a, 20a, 19b and 92 (Network 1) are part of the cluster 17 ~ 92 which was shown to be up-regulated during the clonal expansion step of adipocyte differentiation of 3T3-L1 cells [50]. Over-expression of cluster 17~92 accelerated adipocyte differentiation and triglyceride accumulation by targeting and negatively regulating the gene coding tumor suppressor retinoblastoma-like 2 (p130) [50]. miR-103 and miR-107 were found to be up-regulated in adipocyte differentiation [51] and have been reported to play a role in the regulation of insulin sensitivity [52]. In this context it is likely that Network 1 is involved in the regulation of the development and metabolism within bovine adipose tissue. Similarly, Network 2 has miRNAs known to regulate adipogenesis, such as the highly expressed let-7 family which has been reported to regulate the transition from clonal expansion to terminal differentiation of adipocytes [53]. The identification of let-7a, let-7b, let-7d, let-7 g, let-7f, and miR-98 in Network 2 also gives credibility to the identification of miRNAs co-expression by Relevant Network Analysis as all miRNAs cited above are members of the let-7 family of miRNAs. The smaller networks (4, 5, and 6) may play a role in the regulation of bovine adipose tissue in more limited ways as they were poorly conserved and some were bovine specific (miR-2881, -2328, and 2305). Therefore, these miRNA regulatory networks may contribute to species-specific metabolic characteristics in adipose tissue of bovines or other ruminants as previous studies have pinpointed that bovine adipogenesis is different than that of other species including human and mice [54,55]. Functional studies and experimental validation of target genes of bovine specific miRNAs may shed some light on their specific roles in the function of bovine adipose tissue. Many other AT core miRNAs (n = 33) that were not included in the relevant networks were also organized in clusters suggesting that their expression might also be coordinated with other members of the cluster.

Coordinated expression does not only occur with miRNAs, but intronic miRNAs expression may also be coordinated with host genes [56]. This study revealed that 39% of core adipose miRNAs were coded within introns of host genes (Figure 2) involved in a multitude of cellular and molecular functions (Figure 8). Defining the function of these host genes may help identify the regulatory role of intronic miRNAs, as they may assist the expression/function of the host gene [57] or be involved in the same biological process. In the context of adipose tissue, five intronic miRNAs were identified as being associated with genes potentially involved in lipid metabolism (Table 1). In fact, some of these miRNAs have already been reported to play a role in adipogenesis or lipid metabolism including miR-378 which was found up-regulated in steers with high levels of subcutaneous fat [25]. This miRNA has also been reported to increase the size of lipid droplets in mice ST2 cells when over-expressed [58]. Similarly, miR-33a was found to be involved in the regulation of β-oxidation of fatty acids, cholesterol homeostasis and insulin signalling [59]. Interestingly, anti-miR-33 therapy is under investigation as a promising method to treat cardiometabolic diseases [60]. miR-33a expression in subcutaneous adipose tissue was significantly correlated with its host gene *SREBF2* (Table 2), a transcription factor that controls the expression of other genes involved with cholesterol metabolism [61]. These findings are in agreement with findings that show coordinated expression of miR-33a and *SREBF2* over a range of tissue types [62]. miR-1281 also showed a coordinated expression pattern with its host gene (*EP300*). It is possible that miR-1281 might play a role in adipogenesis as EP300 is a transcriptional co-activator of CCAAT/enhancer binding protein α (C/EBPα) [63], which is a critical transcription factor for adipocyte differentiation [64]. Expression of miR-378 and miR-151-5p did not correlate with expression of their host genes, but this observation does not rule out the possibility that coordinated expression could still occur. For instance, miR-378 in bovine is coded in two different genomic locations, one is located in an intron of *PPARGC1B* gene in chromosome 7 (precursor miR-378-1) and the other in an intergenic region in chromosome 4 (precursor miR-378-2). Therefore, *PPARGC1B* expression is not the only source for miR-378, which may explain why a significant correlation was not found between miR-378 and *PPARGC1B* levels. PTK2 is a signaling molecule that can promote cell motility [65] and its expression has been reported to be coordinated with miR-151 in hepatocellular carcinoma tissue samples [66]. This could be why in adipose tissue of uniform and healthy steers the coordinated expression between miR-151-5p and *PTK2* was not observed. Others have shown that coordinated expression between intronic miRNAs and host gene does not always occur as intronic miRNAs can also be independently transcribed from host genes [67,68].

The group of poorly conserved miRNAs identified in this study includes a subset termed as bovine specific miRNAs, which represent miRNAs with a seed sequence unique to *Bos taurus*. They accounted for approximately 15% of the bovine core adipose miRNAs and were typically non-clustered (Table 3). miRNAs are rarely lost when an animal lineage acquires them [69], therefore bovine specific miRNAs are likely to be evolutionary younger than highly conserved miRNAs as they are not found in other mammalian groups such as primates or rodents. The species specificity of these miRNAs suggests that they may perform a regulatory function that is unique to adipose tissue metabolism in *Bos taurus* or other ruminants. In general, species specific miRNAs tend to be less expressed than highly conserved miRNAs, but bovine specific miR-2478 was the most highly expressed miRNA in all animals from our study suggesting it has an established role in bovine adipose tissue. In addition, miRNAs highly expressed are reported to be under a high selective pressure to maintain their sequence unaltered in comparison to lowly expressed miRNAs that are rapidly evolving with frequent mutations [44]. Analysis of predicted targets for bovine specific miRNA revealed that 17 out of 23 of these core adipose miRNAs may be involved in different aspects of adipose tissue energy balance by targeting genes involved in lipid metabolism, carbohydrate metabolism and/or energy production (Figure 9).

The importance of miRNAs is becoming more evident in the regulation of every biological process in mammals. Understanding how miRNAs influence adipogenesis and fat metabolism is fundamental to develop strategies to manipulate adiposity in beef cattle. Progress has been made in identifying quantitative trait loci (QTL) [70] and genes for economically important traits in cattle. miRNAs add an extra dimension of complexity to the regulation of fat metabolism and adipogenes, therefore; they should be incorporated to genetic studies in order to better understand how complex traits are controlled. Future studies investigating the dynamics of miRNAs in adipose tissue might consider RNA-seq to further explore novel miRNAs as microarrays are not able to profile the expression of unknown miRNAs. High throughput sequencing studies focussing on miRNAs in bovine adipose tissue are still lacking, but a recent study in swine identified a total of 409 unique miRNAs in subcutaneous and visceral fat depots [71] which suggests that there are still many unknown miRNAs expressed in bovine adipose tissue that our microarray study was not able to detect. Another limitation that microarray technology might present for studies comparing the expression of different miRNA species is that signal intensity from hybridization might be affected by factors such as probe sequence (GC%) [72,73].

## Conclusions

In conclusion, the core adipose miRNAs are widely spread on bovine chromosomes, and genomic context features such as miRNA organization in clusters and miRNA evolutionary conservation were associated with their expression and quantity of predicted targets. Core adipose miRNAs are likely to work collectively as their expression revealed that 34 of them are part of six regulatory networks, with each displaying a unique coordinated behavior. Another instance of coordinated expression was identified between two intronic miRNAs and their host genes involved in lipid metabolism (miR-33a/*SREBF2* and miR-1281/*EP300*), suggesting these miRNAs regulate the lipid metabolism pathway that involves their host genes. The specific functions of miRNAs are largely unknown, especially for species specific miRNAs. The bovine specific miRNAs expressed in all animals accounted for almost 15% of the core adipose miRNAs. They are likely to be involved in aspects of adipose tissue metabolism that are unique to bovines and or ruminants with the predicted targets suggesting that 17 of them play a role in regulating the energetic balance of bovine adipose tissue. The results obtained in this study expand our understanding on miRNA functions and behaviour in bovine adipose tissue which might help the development in the future of new strategies to manipulate adiposity in beef cattle improving meat quality and animals productivity.

## Methods

### Animal study and sample collections

A total of eight, 12 month old British-continental crossbred beef steers were housed in individual pens at the Lethbridge Research Centre. Steers were selected based on similar body weight (452 ± 22 kg) and offered either a low fat diet containing 2.7% fat, (Control group, n = 4) or a high fat diet containing 7.1% fat. (High fat group, n = 4) *ad libitum*. Fat content of the diet was increased by including 10.0% flaxseed in the diet. Steers had free access to water and diets were fed for 14 weeks until steers were slaughtered at about 15.5 months of age. Throughout the experiment several performance measures were recorded including body weight gain, feed intake and feed conversion. Carcass traits including cutability, backfat thickness and adipocyte size were also recorded and reported previously [74,75]. Subcutaneous fat was collected from each steer through biopsy at three six-week intervals (12, 13.5 and 15 months). The first biopsy collection (0.2-0.5 g) was performed at 15 cm to the left of the last thoracic vertebrae of each steer. Subsequent biopsies were performed within the same area but 4 and 8 cm left of the scar from the initial biopsy. Subcutaneous fat (backfat) samples were immediately frozen in liquid nitrogen, and stored at - 80°C until analyzed. The animal study was approved by the Animal Care Committee of Lethbridge Research Centre, Agriculture Agri-food Canada with ACC# 0930.

### RNA extraction

Total RNA extraction was performed by homogenizing the fat tissue samples with TRIZOL® (TRI reagent, Invitrogen, Carlsbad, CA, USA) following the manufacturer’s instructions for samples with high fat content. The concentration of total RNA was measured using the NANODROP® spectrophotometer ND-1000 (Thermo Scientific, Waltham, MA, US) and RNA integrity was measured using the Agilent 2100 BIOANALYZER® (Agilent Technologies Deutschland GmbH, Waldbronn, Germany). RNA with integrity number (RIN) >7.8 and concentration > 200 ng/μl was used for miRNA microarray and qRT-PCR analysis.

### Microarray analysis

miRNA profiling of subcutaneous adipose tissue samples (n = 24) at three different time points (12, 13.5, and 15 months) from 8 cattle (4 fed each of the two diets) was performed. AGILENT 8 × 15 K miRNA array V3 (Agilent Technologies, Santa Clara, CA, USA) was customized to profile 672 bovine miRNAs based on the miRBase (Release 15). In brief, total RNA (100 ng) was firstly labeled with the AGILENT miRNA Complete Labeling and Hyb Kit (Version 2.1) by dephosphorylation with calf intestinal phosphatase, followed by denaturing and ligation with Cyanine3-pCp at the 3′ end. The labeled RNA was hybridized with array slides with hybridization buffer and 10X GE blocking agent, and incubated at 55°C for ~20 hours. Finally, the arrays were washed with GE buffers and scanned at 5 μM resolution on an Agilent G2565CA High Resolution Scanner (Agilent Technologies). Data were processed through Agilent’s Feature Extraction software version 10.7.3.1 and the data was normalized to the 75th percentile using GeneSpring GX 11.5 (Agilent Technologies).

### miRNA genomic context and conservation status analysis

Genomic context characteristics of the core bovine adipose miRNA genes and miRNA conservation status were obtained using online bioinformatics search tools. Bovine miRNA organization was classified as clustered or unclustered using the cluster search tool from miRBase (http://www.mirbase.org/search.shtml) considering clustered miRNAs as those with inter-miRNA distance of less than 10 kb and within the same DNA strand [76]. miRNAs on bovine genome were classified according to their location as intronic (located in introns of genes), intergenic (located in regions outside transcription units), exonic (overlap an exon of a gene) and mirtron (its pre-miRNA sequence represents the entire length of an intron) [38]. miRNA genomic location was searched in the cow genome assembly UMD3.1 using the NCBI Map Viewer (http://www.ncbi.nlm.nih.gov/mapview/) and Ensemble genome browser (http://uswest.ensembl.org/index.html). Core adipose miRNAs were classified according to their conservation status as highly conserved, conserved or poorly conserved by searching the TargetScan 6.2 database for conservation of miRNA families (http://www.targetscan.org/cgi-bin/targetscan/mirna_families.cgi?db=vert_61) [28].

### Bioinformatics analysis

miRNA relevant networks were created by connecting adipose core miRNAs according to the correlation (R^2^ ≥ 0.95) of their expression over 24 samples using Relevance Networks tool [77] from the Multiple Array Viewer from Multi Experiment Viewer software (v.4.8) [78]. Prediction of target genes was performed for each core adipose miRNA using TargetScan 6.2 for mammals and customized by species (cow/Bos taurus) (http://www.targetscan.org/vert_61/). Functional analysis of genes hosting adipose core miRNAs located inside their introns was performed through IPA Core Analysis (Ingenuity® Systems, http://www.ingenuity.com). The same method was used for the functional analysis of miRNA predicted targets. Right-tailed Fisher’s exact test was used to calculate a p-value determining the probability that each biological function was relevant to the assigned data set with significance designated at p < 0.05.

### Dataset

All the microarray data in this study are in compliance to MIAME guidelines and the data have been deposited in the publicly available NCBI’s Gene Expression Omnibus Database (http://www.ncbi.nlm.nih.gov/geo/). The data are accessible through GEO Series accession number GSE50489 http://www.ncbi.nlm.nih.gov/geo/query/acc.cgi?acc=GSE50489.

### miRNA and gene expression validation by qRT-PCR

Candidate intronic miRNAs were selected for qRT-PCR validation based on the functional analysis of their host genes. Genes associated with lipid metabolism and their intronic miRNA were selected for qRT-PCR. miRNA expression was carried out with TAQMAN® miRNA assays according to the manufacturer’s recommendation (Applied Biosystems, Foster City, CA, USA). Briefly, cDNAs were reversely transcribed from 10 ng of total RNA using 5X specific miRNA RT primer and were amplified using a 20X TAQMAN® miRNA assay. Fluorescence signal was detected with an ABI STEPONEPLUS Real-time PCR System detector® (Applied Biosystems). In order to asses gene expression, first strand was obtained from total RNA for each sample using random primers and reverse transcription reagents (Invitrogen, Carlsbad, CA, USA) according to manufacturer’s guidelines. Each PCR reaction (20 μL) consisted of 2 ng of template cDNA, 2× SYBR Green I Master Mix buffer (10 μL, Applied Biosystems, Foster City, CA), and 300 n*M* forward and reverse primers. Fluorescence signal was detected with an ABI STEPONEPLUS Real-time PCR System detector® (Applied Biosystems) using the following conditions: 2 min at 50°C, 10 min at 95°C, 40 cycles of 15 s at 95°C and 1 min at 60°C.

A total of 12 samples from six steers (3 from each diet) at two different ages (12 and 15 months) were used for qRT-PCR analysis with a total of 3 technical replicates per reaction for both intronic miRNA and host genes. bta-miR-181a was selected as the reference for miRNA analysis while beta-actin was used as reference for gene expression in this study due to their uniform expression among all samples, with coefficients of variation of 0.029 and 0.044 respectively. The sequences of primers used for qRT-PCR in this study are shown in Additional file 5. Gene expression was analyzed by relative quantification (delta delta Ct method).

### Statistical analysis

The means of miRNA expression and predicted targets according to miRNA conservation status (highly conserved vs. conserved vs. poorly conserved) were analysed by one-way ANOVA and compared by Tukey’s test. Comparisons of means involving miRNA organization (clustered vs. non-clustered miRNAs) and miRNA location (intergenic vs. intronic miRNAs) were performed by a two tailed t-test. The miRNA expressions from qRT-PCR were tested for normality and equal variances, and submitted to an one-way ANOVA and the means were compared by Tukey’s test. Differences were considered statistically different at p < 0.05 and analyses were performed with SAS software (v.9.0).

## Competing interests

The authors declare that they have no competing interests.

## Authors’ contributions

All authors contributed extensively to the work presented in this paper. JMR, WJ, MH, TM, and LLG designed the experiment. JMR, WJ, TM, performed the experiment. JMR, WJ and LLG analyzed the data and wrote the manuscript. MH, TM contributed to revision of the original manuscript. LLG had primary responsibility for final content. All authors read and approved the final manuscript.

## Supplementary Material

Additional file 1 Genomic context, conservation and expression of AT core miRNAs. Click here for file

Additional file 2 Frequency histogram of all pairwise correlations among AT core miRNAs. Click here for file

Additional file 3 Hierarchical dendogram of the expression of all 155 AT core miRNAs. Click here for file

Additional file 4 Molecular and cellular functions of predicted targets of bovine specific miRNAs. Click here for file

Additional file 5 **Primer sequences for qRT-PCR.** Click here for file
