# Supplementary figures and images for: MicroRNAs in bovine adipogenesis: genomic context, expression and function
Source: BMC Genomics. 2014 Feb 18;15:137. doi: 10.1186/1471-2164-15-137 (PMC3930007; doi:10.1186/1471-2164-15-137)

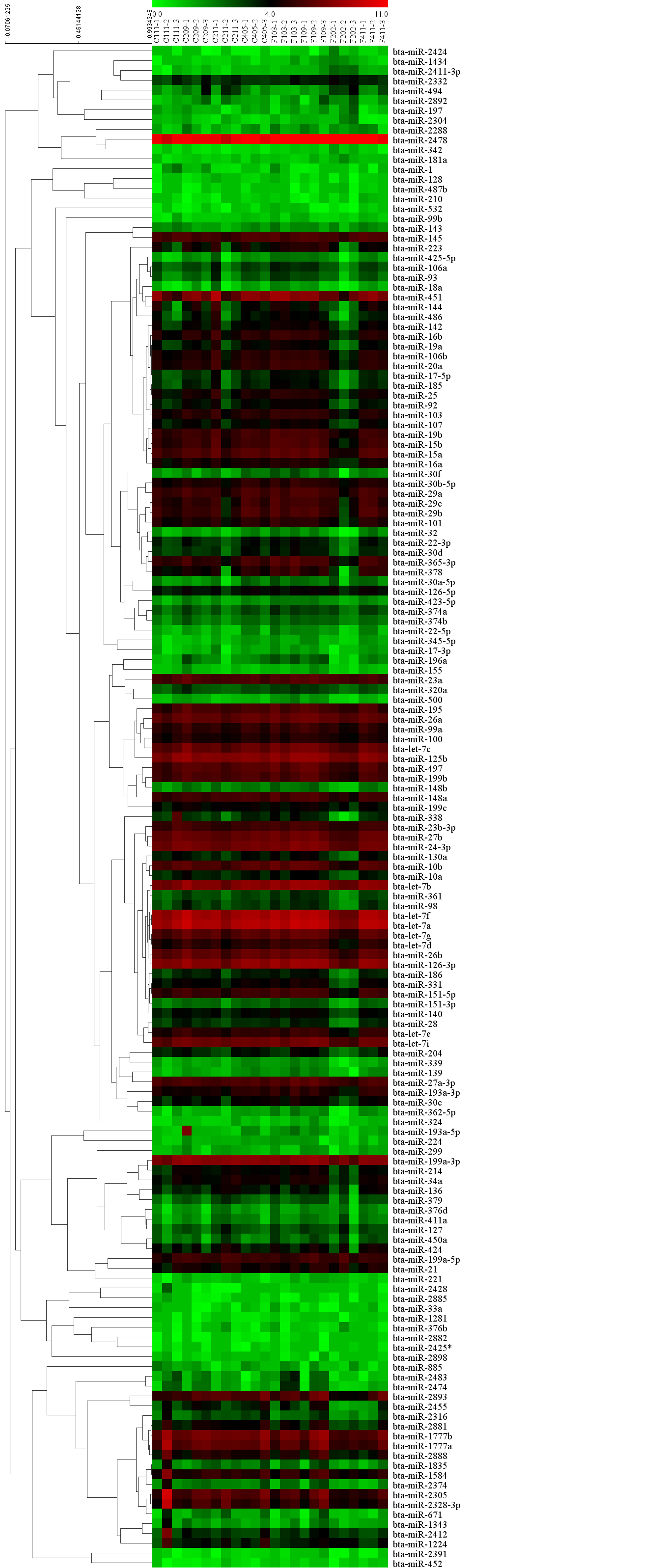

Supplement: Additional file 3 — Hierarchical dendogram of the expression of all 155 AT core miRNAs. [file 1471-2164-15-137-S3.tiff]
