# Supplementary material for: MicroRNAs in bovine adipogenesis: genomic context, expression and function
Source: BMC Genomics. 2014 Feb 18;15:137. doi: 10.1186/1471-2164-15-137 (PMC3930007; doi:10.1186/1471-2164-15-137)
Supplement: Additional file 5 — Primer sequences for qRT-PCR. [file 1471-2164-15-137-S5.docx]

| **Table S3. Primer sequences for qRT-PCR** | | | |
| --- | --- | --- | --- |
| **Gene** | **Description** | **Direction** | **Sequence 5’ - 3’** |
| ACTB | Beta-actin | Forward | CTAGGCACCAGGGCGTAA |
|  |  | Reverse | CCACACGGAGCTCGTTGTA |
| EP300 | E1A binding protein p300 | Forward | GCCTTCGAAGAGATCGATGGT |
|  |  | Reverse | TACTCTCCTCTGGTTGGGCG |
| PPARGC1B | peroxisome proliferator-activated receptor gamma, coactivator 1 beta | Forward | CGGCCTTGTGTCAAGATGGA |
|  |  | Reverse | AGGTGAGGTGCTTGTGTAGC |
| PTK2 | protein tyrosine kinase 2 | Forward | CCAAATGGAGCCAGTGAACCT |
|  |  | Reverse | AAGCACGTGGCCTGCTATG |
| SREBF2 | sterol regulatory element binding transcription factor 2 | Forward | GGGAGACCTACCACGCATC |
|  |  | Reverse | GCTTCATGCAGGAACACCTT |
